# Response to ‘Comment on ‘Domestic light at night and breast cancer risk: a prospective analysis of 105 000 UK women in the Generations Study”

**DOI:** 10.1038/s41416-018-0177-8

**Published:** 2018-10-17

**Authors:** Louise E Johns, Michael E Jones, Minouk J Schoemaker, Emily McFadden, Alan Ashworth, Anthony J Swerdlow

**Affiliations:** 1 Division of Genetics and Epidemiology, The Institute of Cancer Research, London, SM2 5NG UK; 2 Division of Breast Cancer Research, The Institute of Cancer Research, London, SW3 6JB UK; 3 Breast Cancer Now Research Centre at The Institute of Cancer Research, London, SW3 6JB UK; 4 Division of Molecular Pathology, The Institute of Cancer Research, London, SW7 3RP UK; 5 Present Address: Nuffield Department of Primary Care Health Sciences, University of Oxford, Oxford, OX2 6GG UK; 6 Present Address: UCSF Helen Diller Family Comprehensive Cancer Center, San Francisco, CA 94158 USA

We thank the author for their comments on our article.^1^ The questions we asked about light in the bedroom at night^2^ were very similar to those used by Stevens in a case–control study of breast cancer.^3^ It seems curious that when these measures of exposure showed evidence of an association in that study, they were considered to give “evidence that indicators of exposure to light at night may be associated with the risk of developing breast cancer“,^3^ but, apparently, when similar measures of exposure do not show an association they “yield no evidence one way or the other” and are as useful as assessing exposure “on the basis of the flip of a coin”.^1^

As in almost all questionnaire epidemiology, the questions give subjective and imperfect measures of exposure, but this does not make them “no evidence”—they are another brick in an imperfect, hard-to-build, wall, in trying to assess the light at night hypothesis.

## References

[1] Stevens Richard G. (2018). Comment on ‘Domestic light at night and breast cancer risk: a prospective analysis of 105 000 UK women in the Generations Study’. British Journal of Cancer.

[2] Johns LE (2018). Domestic light at night and breast cancer risk: a prospective analysis of 105 000 UK women in the Generations Study. Br. J. Cancer.

[3] Davis S, Mirick DK, Stevens RG (2001). Night shift work, light at night, and risk of breast cancer. J. Natl. Cancer Inst..

